# Effectiveness of COVID‐19 Vaccination in Reducing Severity Among SARS‐CoV‐2 Infected Patients: A Prospective Study From Iraq

**DOI:** 10.1002/hsr2.71768

**Published:** 2026-01-26

**Authors:** Ahmed Hakim Al‐Obaidi, Marwan Majeed Ibrahim, Abdul Hameed Abdul Majeed Al‐Qaseer

**Affiliations:** ^1^ College of Medicine, Department of Internal Medicine Mustansiriya University Baghdad Iraq

**Keywords:** ARDS, COVID‐19, mortality rate, vaccine

## Abstract

**Background:**

Coronavirus disease 2019 (COVID‐19) is a lethal global pandemic that originated in Wuhan, China at the end of 2019. As with other viral infectious diseases, the introduction of an effective vaccine is crucial for stopping the spread of the pandemic.

In Iraq the COVID‐19 vaccination campaign started on March 2, 2021.

**Aim of the Study:**

To determine the influence of the vaccine on new virus infection in Baghdad in terms of its effectiveness on the illness, hospitalization, and mortality.

**Methods:**

A prospective observational study of patients with SARS‐CoV‐2 infection who were had a newly positive Real time PCR testing for SARS‐CoV‐2 at Al‐Yarmouk Hospital or Al‐Adel Primary Health Care Center, and correlate their symptoms to their state of vaccination.

**Results:**

A total of 539 patients tested positive for COVID‐19 via PCR (328 females and 211 males). Among these, 265 patients (49.2%) were vaccinated, while 274 (50.8%) were not. Being vaccinated and receiving the full doses showed the strongest association with decreased disease severity, reduced hospitalization rates, and lower mortality rates (*p* < 0.05 for all). The type of vaccination was significantly correlated with disease severity (*p* < 0.001), with more severe or critical symptoms reported in patients vaccinated with BBIBP‐CorV compared to those vaccinated with BNT162b2 or ChAdOx1 nCoV‐19 (AZD1222). However, no correlation was found with hospitalization or mortality rates.

**Conclusion:**

COVID‐19 vaccination, while not preventing infection, markedly reduced disease severity and mortality—especially with full dosing and among high‐risk groups. The differential effectiveness observed among vaccine types highlights the importance of optimizing vaccine strategies based on real‐world outcomes, particularly in resource‐constrained settings like Iraq.

## 1 Introduction

Coronavirus (CoV) can cause a range of illnesses, from mild flu‐like symptoms to severe pulmonary infections [1, 2]. The current novel CoV illness, also known as SARS‐CoV‐2 and COVID‐19, poses a serious threat to human well‐being [3]. The COVID‐19 infection began in China at the end of 2019 and later spread to other parts of Asia [4, 5]. As of January 1, 2023, there have been 6,672,752 COVID‐19 deaths worldwide [6]. In Iraq, the first COVID‐19 case was identified on February 24, 2020, in Najaf, located 180 km south of Baghdad [7].

Natural herd immunity levels in the population were relatively low, ranging from 1% to 10% across various countries and regions, according to studies conducted in multiple locations, including Switzerland, Spain, the United States, and Brazil, particularly in areas with high infection rates [8, 9]. The development of COVID‐19 vaccines relies on different strategies, depending on how the antigen is presented to the immune system. One approach uses inactivated viral proteins produced in vitro, which are introduced directly into the body to trigger an immune response. Another strategy involves antigens expressed in vivo through viral vectors that deliver genetic material into host cells [10, 11, 12]. Both methods can induce neutralizing antibodies that help protect the host from SARS‐CoV‐2 infection. In August 2021, the FDA declared the first approval of a COVID‐19 vaccine, which was the BNT162b2 COVID‐19 Vaccine, primarily for individuals aged 12 and older [13].

Results from large efficacy trials indicate the BNT162b2 vaccine was effective in over 90% of symptomatic cases [14]. Two doses of the vaccine were recommended to be administered intramuscularly 21 days apart [13]. The Ministry of Health signed the COVAX convention to import COVID‐19 vaccines and at March 2021, Iraq started its vaccination program. It received 50,000 BBIBP‐CorV vaccine doses, then received ChAdOx1 nCoV‐19 (AZD1222) vaccines through the UN's vaccination program at the end of the same month. By mid‐April 2021, the BNT162b2 vaccine arrived in the country. These were the three vaccines approved for use in Iraq.

While many global studies have assessed the efficacy of COVID‐19 vaccines in reducing infection and mortality, limited data are available from low‐ and middle‐income countries (LMICs), particularly in the Middle East, on how vaccination influences the severity of disease, hospitalization, and outcomes across different vaccine types. Moreover, few studies have evaluated the real‐world effectiveness of these vaccines in Iraqi populations where vaccine accessibility, public acceptance, and variant prevalence may differ from high‐income settings. This study aims to address this knowledge gap by evaluating the impact of vaccination on newly diagnosed SARS‐CoV‐2 cases in Baghdad, focusing on disease severity, hospitalization, and mortality rates.

## 2 Patients and Methods

### 2.1 Study Design

This is a Prospective Comparative Observational Study that follows the STROBE (Strengthening the Reporting of Observational Studies in Epidemiology) guidelines for reporting observational research [15]. Ethical approval for this study was obtained from the Department of Medicine, College of Medicine, Mustansiriyah University, Baghdad, Iraq. Permission was granted for the academic use of patient records collected at Al‐Yarmouk Teaching Hospital. The study was conducted in accordance with institutional ethical guidelines and regulations.

A descriptive comparative follow‐up study of adult patients with newly diagnosed SARS‐CoV‐2, confirmed by PCR throat sampling, who were seen or registered at Al‐Adel Primary Health Care Center for Family Medicine, or admitted to Al‐Yarmouk Teaching Hospital in Baghdad, from January 1, 2022, to January 1, 2023. A random sample of subjects who tested positive for COVID‐19 via PCR throat sampling during this period, regardless of their symptoms, was monitored throughout their illness or hospital stay. These individuals were invited to participate and followed until their recovery was announced, either 14 days after the PCR result, upon hospital discharge, or in the case of death.

### 2.2 Definition of Variables

In this study, a range of variables were considered to assess the relationship between COVID‐19 vaccination status and clinical outcomes among infected patients. Personal demographic factors included age and gender. Only adult patients aged 18 years and above were eligible for inclusion. For analytical purposes, age was categorized into three groups: 18–30 years, 31–60 years, and over 60 years. Gender was recorded as either male or female.

Disease‐related variables included the diagnosis of SARS‐CoV‐2 infection confirmed by a positive real‐time PCR test from throat swab samples. The primary clinical outcomes of interest were the severity of illness (categorized as asymptomatic, mild, severe, or critical), the site of isolation or care (home isolation, general ward admission, or intensive care unit), and the prognosis (recovery or death).

Vaccination‐related variables encompassed the patient's vaccination status, the type of vaccine received, and the number of doses administered. Patients were classified as either vaccinated or non‐vaccinated. Among the vaccinated cohort, three types of vaccines were included: BNT162b2 (Pfizer‐BioNTech), ChAdOx1 nCoV‐19 (AZD1222) (Oxford–AstraZeneca), and BBIBP‐CorV (Sinopharm‐BBIBP). Vaccination status was further categorized based on the number of doses received: patients who had received two or more doses were considered fully vaccinated. In contrast, those who had received only a single dose were classified as partially vaccinated.

Vaccination data were obtained from hospital admission records, primary health care vaccination center logs, and patients' official vaccination cards, which also detailed the vaccine type and dose count.

All vaccinated participants in this study received homologous vaccine schedules, meaning all doses were from the same manufacturer. No cases of heterologous vaccination (mixed vaccine types) were recorded.

The effectiveness of vaccination was assessed by comparing clinical outcomes—specifically, the severity of illness, hospitalization rate, and mortality—between vaccinated and unvaccinated individuals. The underlying assumption was that vaccination may not prevent infection entirely, but could reduce the severity of the disease and related complications. This approach aligns with established methods in vaccine effectiveness research, where outcomes such as hospitalization and mortality are often used as primary endpoints.

### 2.3 Sampling Technique

A simple random sampling method was employed to select participants from the registry of PCR‐confirmed COVID‐19 cases at Al‐Yarmouk Teaching Hospital and Al‐Adel Primary Health Care Center. Eligible patients were assigned serial numbers, and random numbers were generated using SPSS software to ensure unbiased selection and minimize sampling error.

### 2.4 Disease Severity Classification

The clinical severity of COVID‐19 among study participants was categorized into four groups—asymptomatic, mild, severe, and critical—based on standardized criteria adapted from the World Health Organization (WHO) clinical management guidelines [16].

Patients classified as asymptomatic were those who tested positive for SARS‐CoV‐2 by real‐time PCR but exhibited no clinical symptoms during the course of illness. The mild category included patients who reported symptoms such as fever, cough, sore throat, malaise, headache, or muscle pain, yet showed no clinical or radiological evidence of pneumonia or hypoxia.

Patients with severe COVID‐19 were defined as those presenting with respiratory distress (respiratory rate greater than 30 breaths per minute), oxygen saturation below 93% on room air, or clinical signs of pneumonia with or without imaging confirmation. The critical group comprised patients who developed complications such as respiratory failure requiring mechanical ventilation, septic shock, or multi‐organ dysfunction necessitating intensive care unit (ICU) admission.

### 2.5 Statistical Analysis

The Statistical Package for Social Sciences (SPSS) software version 22.0 was used to analyze the questionnaire data—software for Windows (SPSS Inc., Chicago, IL, USA). In the case of continuous variables, data are presented as means and standard deviations (SDs); for categorical variables, data are presented as absolute numbers and percentages. The *χ* ^2^ test was used to compare categorical variables, and independent‐samples *t*‐tests were used for continuous variables where appropriate. All statistical tests were two‐sided. Significance was defined as a *p* value < 0.05.

## 3 Results

The general characteristics of all patients are given in (Table 1) including: age, gender, vaccination status, type of vaccination, number of vaccine doses, clinical presentations, site of isolation, and prognosis of the illness. A total of 539 patients with newly discovered positive SARS‐COV2 PCR of throat swap sampling were included in this study. Their mean age 42.9 ± 16.2 years. Where the oldest one was of 90 years, and the youngest one was of 18 years. Female patients comprised 60.9% of the study population. Regarding severity of the SARS‐COV2 infection: those with mild symptoms represent the majority with 453 patients (84%), asymptomatic patients were 40 (7.4%), severely symptomatic patients were 42 (7.8%), and finally those with critical symptoms were 4 (0.7%). Home isolated patients compromised a percentage of 95.5% (515 patients), and the rest 4.5% (24) were admitted to hospitals.

**Table 1:** General characteristics of the study patients (*N* 539).

Variable		Mean (SD)	*N* (%)
Age	All	42.4 (16.2)	
	Age 18–30 years		148 (27.5)
	Age 31–60 years		298 (55.3)
	Age > 60 years		93 (17.3)
Gender	Male		211 (39.1)
	Female		328 (60.9)
Symptoms	Asymptomatic		40 (7.4)
	Mild symptoms		453 (84)
	Severe symptoms		42 (7.8)
	Critical		4 (0.7)
Site of isolation and treatment	Home isolation		515 (95.5)
	Ward admission		20 (3.7)
	ICU		4 (7)
Vaccination status	Vaccinated		265 (49.2)
	Non‐vaccinated		274 (50.8)
Type of vaccine	BNT162b2		180 (67.9)
	ChAdOx1 nCoV‐19 (AZD1222)		37 (14)
	BBIBP‐CorV		48 (18.1)
Number of vaccine doses received	Single dose		25 (9.4)
	Two doses		214 (80.8)
	Three doses		26 (9.8)
Prognosis	Recovered		531 (98.5)
	Died		8 (1.5)

A total of 265 patients were vaccinated, accounting for about 49.2% of all patients, while the remaining 274 (50.8%) were unvaccinated. Of the 265 vaccinated patients, the BNT162b2 Pfizer‐BioNTech vaccine was the most commonly administered, with 180 patients (67.9%) receiving it, while 48 patients (18.1%) received the BBIBP‐CorV Sinopharm‐BBIBP vaccine and 37 patients (14%) received the ChAdOx1 nCoV‐19 (AZD1222) Oxford–AstraZeneca vaccine. The majority of vaccinated patients, 80.8% (214), received two doses of the vaccine, while 9.8% (26) received three doses, and those who received only one dose represent 9.4% (25). Regarding patients' final prognosis after contracting the infection, 98.5% (531) were reported to have recovered, while 1.5% (8 patients) died.

In Table 2, we assess the correlation between patients' factors and their symptoms of SARS‐CoV‐2 infection, isolation site, and disease prognosis. There is a significant association with age, featuring a *p* value of 0.005, < 0.0001, and < 0.0001, respectively, for patients over 60 years old, as this age group tends to experience more severe disease, increased hospitalizations, and higher mortality rates compared to younger patients. Gender shows no significant correlation, with *p* values of 0.058, 0.134, and 0.526, respectively.

**Table 2:** Correlation between personal factors and patients' severity of symptoms of SARS‐COV2 infection, site of isolation, and prognosis of the disease.

Factor		Severity		*p* value	Site of isolation		*p* value	Prognosis		*p* value
		Asymptomatic or mild symptoms	Severe or critical symptoms		Home isolation	Ward or ICU		Recovered	Died	
Sex	Male	187	24	**0.058**	197	14	**0.134**	207	4	**0.526**
	Female	306	22		318	10		324	4	
Age	18–30 years	144	4	**0.005**	146	2	**< 0.0001**	148	0	**< 0.0001**
	30–60 years	269	29		284	14		296	2	
	> 60 years	80	13		85	8		87	6	

In Table 3, assessing the correlation between vaccination factors and patients' severity of symptoms of SARS‐CoV2 infection, site of isolation, and prognosis of the disease. The vaccination status was found to significantly affect the severity of the disease, hospitalization, and death rate, with a *p* value of 0.003, 0.017, and 0.037, respectively; where non‐vaccinated patients tend to have more severe symptoms, more hospitalization, and more deaths than vaccinated ones. The type of vaccination was of significant relevance regarding the severity of the disease, with *p* value of 0.00005, where the presentation with severe or critical symptoms was more in BBIBP‐CorV vaccinated patients than those with BNT162b2 or ChAdOx1 nCoV‐19 (AZD1222) vaccines, while it shows no significant correlation with hospitalization or prognosis of the patients with *p* value of 0.106, 0.103, respectively.

**Table 3:** Correlation between vaccination factors and patients' severity of symptoms of SARS‐COV2 infection, site of isolation and prognosis of the disease.

Factor		Severity		*p* value	Site of isolation		*p* value	Prognosis		*p* value
		Asymptomatic or mild symptoms	Severe or critical symptoms		Home isolation	Ward or ICU		Recovered	Died	
Vaccination status	Vaccinated	252	13	**0.003**	260	5	**0.017**	264	1	**0.037**
	Non‐vaccinated	241	33		255	19		267	7	
Type of vaccine	BNT162b2	176	4	**0.00005**	178	2	**0.106**	180	0	**0.103**
	ChAdOx1 nCoV‐19 (AZD1222)	37	0		37	0		37	0	
	BBIBP‐CorV	39	9		45	3		48	1	
Number of vaccine doses	Partially vaccinated	21	4	**0.007**	22	3	**0.00003**	24	1	**0.002**
	Fully vaccinated	231	9		238	2		240	0	

The number of doses of vaccine was significantly correlated to the severity of symptoms, hospitalization, and prognosis with *p* value of 0.007, 0.00003, and 0.002, respectively, where the presentation with severe or critical symptoms, hospitalization, and death were more in those partially vaccinated than those receiving full doses.

Table 4 shows the correlations between vaccination status and severity of the disease, site of isolation, and prognosis in different patients' age groups. There is significant correlation in patients' age group of > 60 years, where those non‐vaccinated patients of this age group tend to have more severe symptoms, more hospitalizations, and more deaths than vaccinated patients of the same age group, *p* value: < 0.001, 0.006, 0.017, respectively. There is no significant correlation in patients of 18–30 years and 31–60 years.

**Table 4:** Correlation between vaccination status and severity of the disease in different patients' age groups.

Age group	Vaccination status	Severity		*p* value	Site of isolation		*p* value	Prognosis		*p* value
		Asymptomatic or mild symptoms	Severe or critical symptoms		Home isolation	Ward or ICU		Recovered	Died	
18–30 years	Vaccinated	62	0	**0.085**	62	0	**0.22**	62	0	
	Non vaccinated	82	4		84	2		86	0	
31–60 years	Vaccinated	134	11	**0.22**	141	4	**0.22**	145	0	**0.168**
	Non vaccinated	135	18		143	10		151	2	
> 60 years	Vaccinated	56	2	**< 0.001**	57	1	**0.006**	57	1	**< 0.017**
	Non vaccinated	24	11		28	7		30	5	

## 4 Discussion

The financial and social life of people and societies has been negatively impacted by the COVID‐19 pandemic, which has also caused significant global morbidity and mortality. The majority of the people are still vulnerable to COVID‐19 infection despite this tragic toll. So, introducing vaccines was a top priority [7]. Unprecedented advances to be made in vaccine introduction, and the delivery of highly effective vaccines has begun. This study reveals that COVID‐19 vaccine had a positive impact on the disease, even if they gave little protection against infection, might greatly lessen severe attack rates, hospitalizations, and so on fatalities.

In accordance with multiple previous studies conducted in China, Spain, and the United Kingdom, we found that older age is a risk factor for developing severe disease. This may be explained by the fact that elderly individuals typically provide a weaker type I interferon (IFN) response to the virus, which can exacerbate a condition where weak or completely suppressed type I IFN responses allow the virus to replicate and cause more tissue damage, subsequently triggering a stronger immunological reaction as the immune system struggles to halt viral replication and to recover affected tissue [16].

We conclude that vaccination provided limited protection against mild to moderate COVID‐19 infection but significantly reduced the likelihood of developing severe or critical disease, hospitalization, and mortality. A finding identical to what is found in a study done in South Africa. While it shows a significant impact of vaccination in decreasing the likelihood of having severe and critical diseases, that is also found in multiple previous studies done in the United States of America, Lebanon, Argentina, and a review from worldwide vaccination campaigns [17]. Also, hospitalization was found to be averted by vaccination in our study, which is identical to previous studies done in the USA, Qatar, and multiple other countries [18]. Vaccination was found to decrease mortality in our study.

The study shows that the BBIBP‐CorV vaccine provides lower protection against severe or critical disease, this was by studies done in Peru and Iran in 2022. It might be due to a lack of its effect on new variations of the virus. Also, a study done in China agrees with that result, where it found that the level of neutralizing antibodies produced 28 days after vaccination with the BBIBP‐CorV vaccine is low, even compared to those produced by previous COVID‐19 infection [19].

Our study's findings on the decreased incidence figures of COVID‐19 severe disease, hospitalization, and death among fully vaccinated patients (3.8% and 0.8%, respectively) compared to partially vaccinated patients (19% and 13%, respectively) align with earlier analyses of the impact of the COVID‐19 vaccine. In the United Kingdom, for instance, an analysis of the National Immunization Management Service and the Coronavirus Clinical Information Network revealed that of the 40,000 cases of COVID‐19 admitted to hospitals, 84% had not received vaccines, 13% had only received a single dose of the vaccine, and 3% had received a booster dose. Additionally, numerous studies conducted in the UK, USA, and Brazil showed that being partially vaccinated (receiving a single dose of the vaccine) offers less protection against severe or critical disease, less reduction in hospitalization, and lower mortality rates [19].

## 5 Limitations

This study has several limitations. One limitation is the unavailability of precise data regarding the time interval between the last vaccine dose and the onset of SARS‐CoV‐2 infection. As this interval may influence the degree of immune protection, future studies should aim to include this variable for more refined analysis. Additionally, circulating SARS‐CoV‐2 variants were not identified, which may have affected vaccine effectiveness. Data were collected from two centers in Baghdad, which may limit generalizability. No patients received heterologous vaccine schedules, and laboratory markers such as antibody titers or viral loads were not assessed. Furthermore, there was a notable imbalance in the number of participants receiving different vaccine types, particularly the predominance of BNT162b2 recipients, which may have introduced bias or limited the strength of comparisons across vaccine groups.

## 6 Conclusions

This study demonstrated that age was the most significant risk factor for severe illness, hospitalization, and mortality due to SARS‐CoV‐2 infection. The findings provide strong evidence that COVID‐19 vaccination substantially reduced the severity of disease, the need for hospitalization, and mortality rates. Full vaccination—defined as receiving two or more doses—offered greater protection than partial vaccination with a single dose. While all vaccine types showed a protective effect, the BNT162b2 and ChAdOx1 nCoV‐19 (AZD1222) vaccines were associated with better outcomes compared to the BBIBP‐CorV vaccine. Notably, the protective benefits of vaccination were more pronounced in older individuals.

Based on these findings, it is recommended to intensify vaccination efforts, particularly among high‐risk groups such as the elderly. Public health strategies should emphasize the importance of completing the full course of vaccination and receiving booster doses as scheduled. Individuals are encouraged to accept any available vaccine offered at certified vaccination centers to maximize community‐level protection.

## Author Contributions

Dr. Ahmed Hakim Al‐Obaidi contributed to the conception and design of the study, data collection, analysis, and manuscript writing. Dr. Marwan Majeed Ibrahim participated in data acquisition, literature review, statistical analysis, and drafting of the results section. Prof. Dr. Abdul Hameed Abdul Majeed Al‐Qaseer supervised the research project, contributed to the interpretation of the results, and provided critical revisions for intellectual content. All authors reviewed and approved the final manuscript.

## Funding

The authors did not receive any specific funding for this study.

## Ethics Statement

The study was approved by the Department of Medicine, College of Medicine, Mustansiriyah University, Baghdad, Iraq.

## Consent

Informed consent was obtained from all participants or their legal guardians.

## Conflicts of Interest

The authors declare no conflicts of interest.

## Transparency Statement

The corresponding author, Marwan Majeed Ibrahim, affirms that this manuscript is an honest, accurate, and transparent account of the study being reported; that no important aspects of the study have been omitted; and that any discrepancies from the study as planned (and, if relevant, registered) have been explained.

## Data Availability

The data sets generated and analyzed during the current study are available from the corresponding author upon reasonable request.
